# Mitigation of noise-induced bias of PET radiomic features

**DOI:** 10.1371/journal.pone.0272643

**Published:** 2022-08-25

**Authors:** Ananthi Somasundaram, David Vállez García, Elisabeth Pfaehler, Joyce van Sluis, Rudi A. J. O. Dierckx, Elisabeth G. E. de Vries, Ronald Boellaard

**Affiliations:** 1 Department of Nuclear Medicine and Molecular Imaging, Medical Imaging Center, University Medical Center Groningen, Groningen, The Netherlands; 2 Department of Radiology and Nuclear Medicine, Amsterdam UMC–Location VU University Medical Center, Amsterdam, The Netherlands; 3 Department of Nuclear Medicine, University Hospital Juelich, Aachen, Germany; 4 Department of Medical Oncology, University Medical Center Groningen, University of Groningen, Groningen, The Netherlands; Los Alamos National Laboratory, UNITED STATES

## Abstract

**Introduction:**

One major challenge in PET radiomics is its sensitivity to noise. Low signal-to-noise ratio (SNR) affects not only the precision but also the accuracy of quantitative metrics extracted from the images resulting in noise-induced bias. This phantom study aims to identify the radiomic features that are robust to noise in terms of precision and accuracy and to explore some methods that might help to correct noise-induced bias.

**Methods:**

A phantom containing three ^18^F-FDG filled 3D printed inserts, reflecting heterogeneous tracer uptake and realistic tumor shapes, was used in the study. The three different phantom inserts were filled and scanned with three different tumor-to-background ratios, simulating a total of nine different tumors. From the 40-minute list-mode data, ten frames each for 5 s, 10 s, 30 s, and 120 s frame duration were reconstructed to generate images with different noise levels. Under these noise conditions, the precision and accuracy of the radiomic features were analyzed using intraclass correlation coefficient (ICC) and similarity distance metric (SDM) respectively. Based on the ICC and SDM values, the radiomic features were categorized into four groups: poor, moderate, good, and excellent precision and accuracy. A “difference image” created by subtracting two statistically equivalent replicate images was used to develop a model to correct the noise-induced bias. Several regression methods (e.g., linear, exponential, sigmoid, and power-law) were tested. The best fitting model was chosen based on Akaike information criteria.

**Results:**

Several radiomic features derived from low SNR images have high repeatability, with 68% of radiomic features having ICC ≥ 0.9 for images with a frame duration of 5 s. However, most features show a systematic bias that correlates with the increase in noise level. Out of 143 features with noise-induced bias, the SDM values were improved based on a regression model (53 features to excellent and 67 to good) indicating that the noise-induced bias of these features can be, at least partially, corrected.

**Conclusion:**

To have a predictive value, radiomic features should reflect tumor characteristics and be minimally affected by noise. The present study has shown that it is possible to correct for noise-induced bias, at least in a subset of the features, using a regression model based on the local image noise estimates.

## Introduction

In oncology, PET is routinely used for cancer diagnosis and staging based on the tumor-node-metastasis (TNM) system and it is increasingly used to assess response to treatment using standard quantitative metrics such as standardized uptake values (SUV_peak_, SUV_mean_, and SUV_max_) and metabolically active tumor volume (MATV) [1–4]. Recently, the focus has shifted to automated extraction of a large number of quantitative features from medical images, called radiomics, which could be used for personalized treatment [5–7].

PET radiomics provide a more comprehensive characterization of the tumor tracer uptake than conventional SUV metrics. Tumor uptake heterogeneity, based on the radiotracer used, may reflect the underlying biological processes. These tumor uptake heterogeneity characteristics can be quantified by radiomics. Radiomics can therefore be used to facilitate personalized medicine by supporting the identification of patient candidates for targeted therapy, providing prognostic information to assess response to therapy, and predicting patient survival outcome [8–10]. Several studies have demonstrated that ^18^F-FDG PET-derived radiomics might have a better predictive value than conventional SUV metrics for a variety of cancer types [11–15].

Treating cancer patients with immunotherapy based on monoclonal antibodies (mAbs) has been shown to have the potential to elicit an effective immune response against tumors, and ^89^Zr-Immuno-PET has demonstrated to be useful in predicting the efficacy of immunotherapy in oncological studies [16]. ^89^Zr-Immuno-PET derived radiomics may have the potential to support personalized immunotherapy by eliminating the limitations caused by tumor heterogeneity [17].

One major challenge in radiomics is its sensitivity to several factors such as image acquisition, reconstruction protocol parameters, segmentation methods, image pre-processing, and noise [18–22]. The variability in many of these factors affecting SUV is also addressed in imaging procedure guidelines and by PET/CT system accreditation programmes [23–25]. These guidelines and accreditations aim to minimize the variability in PET reads between systems and institutes, i.e., to improve reproducibility, in ^18^F-FDG PET. However, in ^89^Zr-Immuno-PET, the low positron yield of ^89^Zr (22.6%) in combination with the low amounts of injected radioactivity (~37 MBq), results in images with a poor signal-to-noise ratio (SNR). And, this low SNR affects the precision (repeatability) and accuracy (bias) of the quantitative metrics extracted from these images [26]. Here, precision is a measure of the statistical variability (for example, standard deviation or coefficient of variation) in radiomics extracted from PET images of the same phantom with identical scan duration. On the other hand, accuracy is a measure of the systematic bias in radiomics due to noise; in other words, it measures the deviation in radiomics extracted from noisy PET images compared to those from an almost noise-free image of the same phantom, where the different noise levels are simulated with changing scan duration.

The current study focuses on the sensitivity of PET radiomics to noise and the effect of noise on their precision and accuracy. This experimental phantom study aims to identify the radiomic features (hereafter referred to as RF) that are (1) robust to noise in terms of precision and accuracy and (2) to explore methods that can correct the noise-induced bias of low accuracy features (the term feature hereafter only refers to radiomic features) provived they have high precision.

## Materials and methods

### 3D printed phantom acquisitions

**Fig 1:**
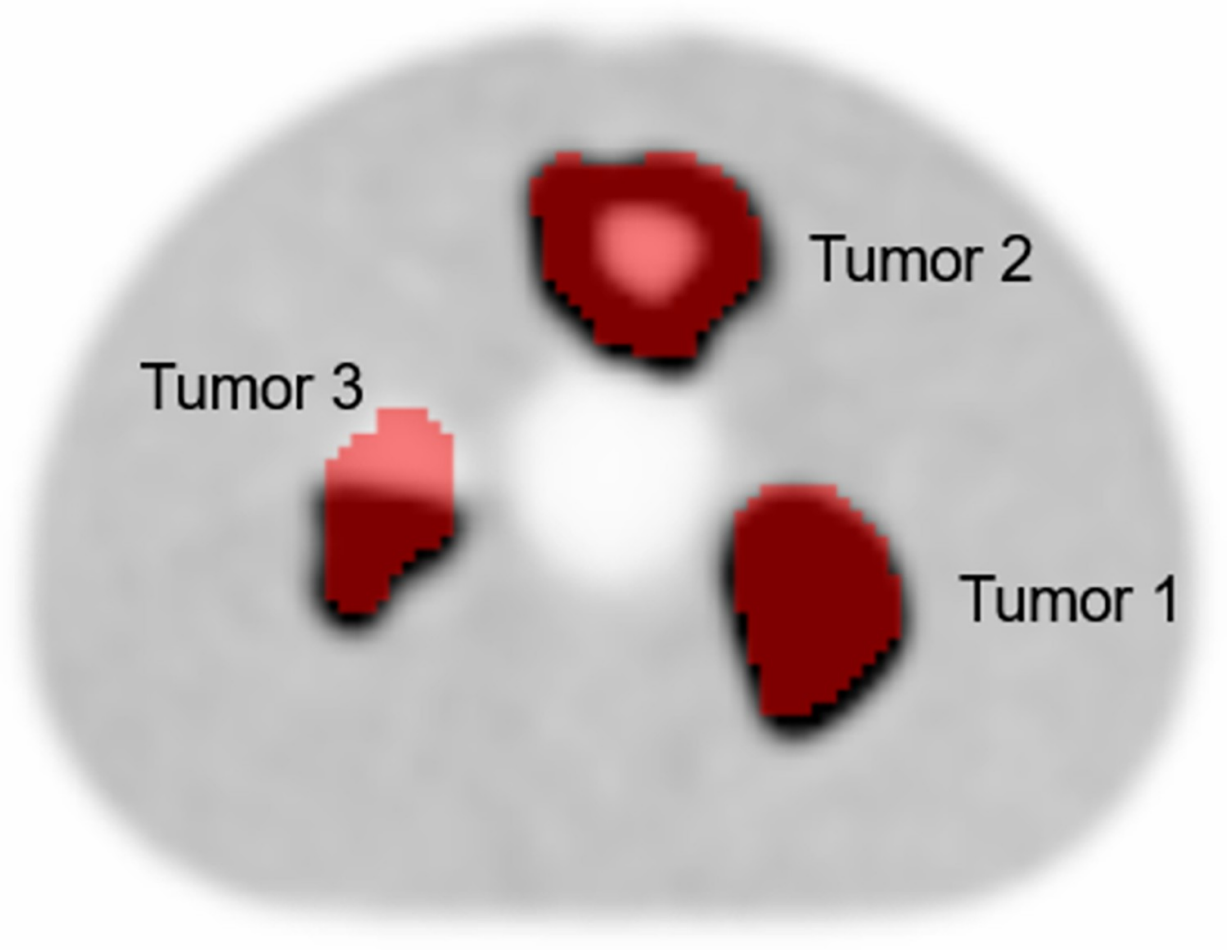
PET image showing phantom with 3D printed inserts reflecting heterogeneous tracer uptake.

A NEMA IQ phantom containing three ^18^F-FDG filled 3D printed inserts was used in the study [19], reflecting heterogeneous tracer uptake and realistic tumor shapes. The phantom inserts were constructed using tumor samples from Non-Small-Cell-Lung-Cancer (NSCLC) patients to obtain characteristics that reflect those seen in patients. Tumor insert 1 (tumor 1) simulates a homogeneous uptake, tumor 2 reflects a tumor with a necrotic core, and tumor 3 represents a heterogeneous uptake by having only one of the two compartments filled with radioactivity (Fig 1). The phantom was scanned with three different tumor-to-background activity concentration ratios (TBR): 10:1, 5:1, and 2.5:1. The tumors were filled, respectively, with activity concentrations of 14.43, 7.17, and 3.65 kBq/ml with a background activity concentration of 1.6, 1.61, and 1.22 kBq/ml, resulting in the intended TBR. Therefore, the three different phantom inserts have been filled and scanned with three different TBR simulating a total of nine different tumors.

A list-mode scan of 40 minutes was acquired for each phantom filling on the Siemens Biograph mCT40 PET/CT system (Siemens Medical Solutions, USA). From the list-mode scans, ten frames each for 5 s, 10 s, 30 s, and 120 s frame duration were reconstructed. The shorter frame durations were used to generate images with different noise levels. The images based on 5 or 10 s frame duration simulate noise levels typically seen in ^89^Zr-Immuno-PET studies, while the images with a frame duration of 120s have noise levels seen with clinical FDG studies. All images were reconstructed using the ordered subset expectation maximization (OSEM) iterative reconstruction method (3 iterations, 21 subsets) with time-of-flight (TOF) and resolution modeling (or point-spread-function, PSF). Two European Association of Nuclear Medicine Research Ltd (EARL) compliant reconstruction protocols EARL1 [24] (using a 6.5 mm full width at half maximum Gaussian filter) and EARL2 [25] (using a 5.0 mm full width at half maximum Gaussian filter) were applied as well. The image matrix size for all images was 256 x 256 x 111 with a voxel size of 3.1819 mm x 3.1819 mm x 2.0 mm. A low-dose CT scan (80 kV, 99 mAs) was used for attenuation correction.

### Segmentation

Volumes of interest (VOI) delineations were made using the in-house software tool ACCURATE [27]. VOIs were manually defined on the low-dose CT images and projected onto the corresponding reconstructed PET images.

### Radiomic feature calculation

Overall, 480 features, belonging to 11 feature groups, were extracted from PET images using the RaCaT tool [28] (version 1.19) as per the Imaging Biomarker Standardization Initiative (IBSI) guidelines [29] (Table 1). The Python packages nibabel [30] (version 2.5.1) and pydicom [31] (version 1.3.0) were used to convert the PET DICOM images and segmented VOI files to NIfTI format. All the features are listed in S1 Table. Since morphological features only depend on the segmentation and not on the PET data, they were excluded from the analysis. Before feature calculation, the images were converted from Bq/ml to SUV resulting in a background SUV equal to 1. The following data extraction settings were used: discretization with a fixed bin width (FBW) of 0.25 SUV [19], resampling to isotropic voxel size of 2 mm using tri-linear interpolation of the images and VOIs [20], and an 8-connected and 26-connected neighborhood for the analysis of the distribution of voxels in 2D and 3D respectively (with the neighborhood consisting of voxels within Chebyshev distance of 1).

**Table 1:** Feature groups with the number of features per group.

Feature group	Number of features
Local intensity	2
Statistics	18
Intensity histogram	24
Intensity volume histogram	6
Grey level co-occurrence (GLCM)	150
Grey level run length (GLRLM)	96
Grey level size zone (GLSZM)	48
Grey level distance zone (GLDZM)	48
Neighbourhood grey tone difference (NGTDM)	15
Neighbouring grey level dependence (NGLDM)	51

### Analysis of radiomic features

All data analysis was performed using Python (version 3.7.6). In this section, the precision and accuracy of the features were analyzed.

### Analysis of precision

The precision or repeatability of the features was analyzed using the intraclass correlation coefficient (ICC). A two-way mixed-effects model was used to evaluate the absolute agreement between the features derived from the ten replicates. ICC was calculated using the R package: irr [32] (version 0.84.1), with the rpy2 package [33] (version 2.9.4) as the Python interface to R.

Based on the ICC values, the features were categorized as having poor (ICC < 0.5), moderate (0.5 ≤ ICC < 0.75), good (0.75 ≤ ICC < 0.9), or excellent (ICC ≥ 0.9) precision.

To study the effect of noise on feature performance, the percentage of features belonging to each of the four categories was calculated per feature group and in total for each of the scan durations.

### Analysis of accuracy

A similarity distance metric (SDM) was used to calculate the distance between the features derived from noisy short frame duration images and the long, nearly noise-free, 40-minute reference scan. Thus, SDM was used to quantify the noise-induced bias of the features. SDM ranges from 0 to 1, and the higher the SDM the better the accuracy, or in other words the closer the noisy scan feature to the reference scan feature.

SDM was calculated using the formula:

$$SDM=\frac{1}{m}\sum_{1}^{m}\frac{\sigma_{l}^{2}}{\frac{1}{n}\sum_{1}^{n}d^{2}(l,s)+\sigma_{l}^{2}}$$

where,

*n* is the number of tumor types (i.e., 9 tumors)

*m* is the number of repeated measurements (i.e., 10 frame repetitions per tumor type)

$\sigma_{l}^{2}$ is the variance between the features of *n* tumor types in the reference scan (*l*)

$d^{2}(l,s)$ is the squared Euclidean distance between the noisy (*s*) and reference scan (*l*) feature values

This metric can be interpreted as the ratio of the variance between the (nine simulated) tumor features in the reference scan to the total variance, where the total variance is the sum of variance between the tumor features in the reference scan and the squared Euclidean distance between the noisy and reference scan feature values. Thus, if a calculated feature is accurate for high noise levels, the squared Euclidean distance between the noisy and reference scan features is negligible when compared to the differences in feature values between the simulated tumors, and this results in an SDM value close to 1.

Based on the SDM values, the features were categorized as having poor (SDM < 0.5), moderate (0.5 ≤ SDM < 0.75), good (0.75 ≤ SDM < 0.9), or excellent (SDM ≥ 0.9) accuracy, in the order from large to negligible noise-induced bias.

To explore the effect of noise on feature accuracy, the percentage of features belonging to each of the 4 categories was calculated per feature group and per frame duration.

### Radiomic feature correctability

*Identification of robust features*. Based on their sensitivity to noise, the features were first classified into the following categories based on ICC and SDM:

1. ICC < 0.9: Low repeatability features. These features were excluded from further analysis due to the low reliability to use them in clinical practice as such.
2. ICC ≥ 0.9 and SDM < 0.9: Potential features, i.e., features with excellent precision but displaying noise-induced bias. These features are potentially useful when corrected for noise-induced bias.
3. ICC ≥ 0.9 and SDM ≥ 0.9: Ideal features. These features have excellent precision and accuracy and can be applied regardless of the noise level of the images.

**Fig 2:**
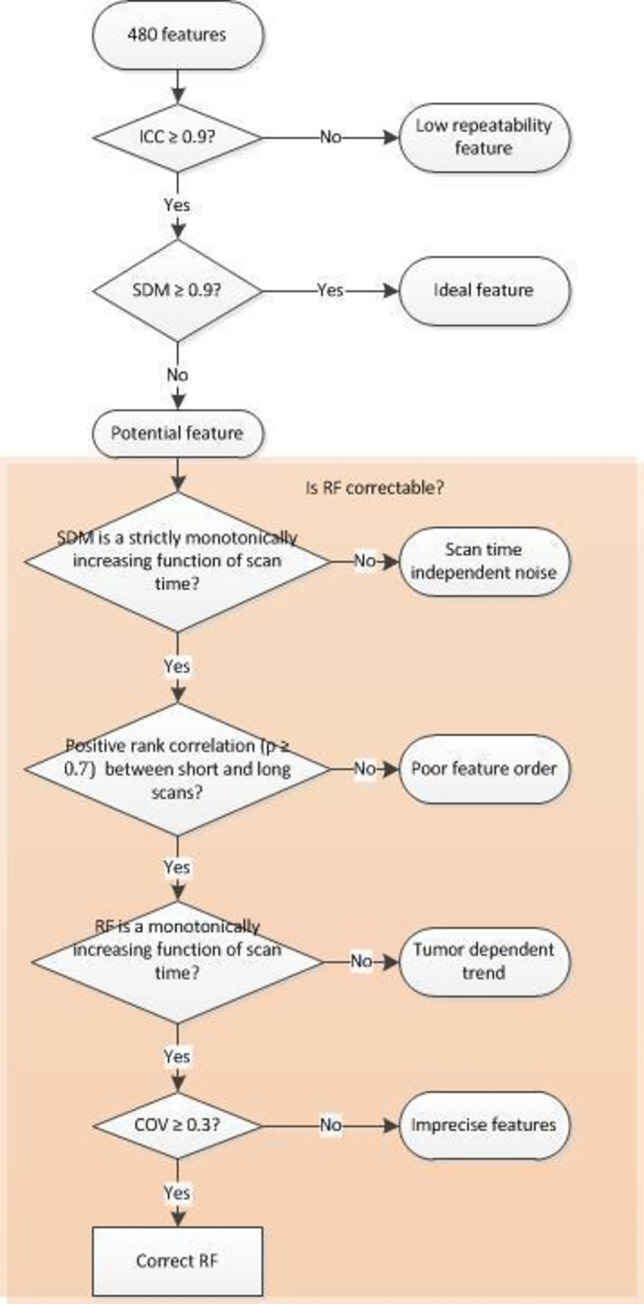
The tests performed to check feature correctability.

*Evaluation of feature correctability*. To evaluate the feasibility to correct the features for noise-induced bias, the behavior of the features at different noise levels was studied and categorized (Fig 2):

1. Monotonic increase of SDM as a function of the noise level. The correlation between the feature accuracy and the scan duration was analyzed using Spearman’s correlation. Only the features with positive correlation (r = 1) were selected.
2. Positive rank correlation between the short and the long scan features. It was assumed that a feature derived from a short scan should correlate with that of a long one to be considered reliable. Since this relationship can be non-linear, the Spearman’s correlation test was used to check if there was a positive correlation between high and low SNR scans. Only a positive correlation was used since a negative correlation would mean that feature order is not maintained between short and long scans. A rank correlation threshold of 0.7 (r ≥ 0.7) was used.
3. Monotonic trend direction of RF as a function of the noise level. With some types of tumors, the features can show a positive bias corresponding with the scan duration, while other tumors can have a negative bias. Features in which the direction of noise-induced bias depends strongly on the underlying uptake distribution will be impossible to correct as in clinical practice this would imply a unique correction (method) for each tumor. We, therefore, restricted the feature correction to only those features that presented the same direction of noise-induced bias across all simulated tumors. This was verified by checking if the feature shows the same monotonic trend direction for all the tumors using Spearman’s correlation test, i.e., either monotonically increasing (r ≥ 0.7) or monotonically decreasing (r ≤ 0.7).
4. Coefficient of variation of the RF. Even if a feature has high ICC, the feature can still have poor precision, i.e., a high coefficient of variation (COV) or standard deviation (SD). Hence, the COV of features at higher noise levels was checked whether it was below 30%, with the purpose to further eliminate imprecise features and restrict the number of features to be corrected.

The features which pass all the aforementioned steps were considered for correction purposes and are hereafter referred to as correctable features.

### Radiomic feature correction

To have an estimate of the local noise, a “difference image” was created by subtracting two statistically equivalent replicate images (frame 1 and frame 2) for each short scan duration. The intensity level in the difference image gives an indication of the local noise in the replicates. Ideally, the difference image should have voxels with an intensity of 0 in the absence of noise. In other words, the lower the intensity values in the difference image, the lower the noise-induced variability in the replicates. The local noise was then characterized by the use of this difference image. Thus, the hypothesis was that the SD and COV of the voxel intensity values in the tumor VOI of the difference image, hereafter referred to as SD_diff_ and COV_diff_ respectively, could be used to characterize local noise. COV_diff_ was computed by dividing SD_diff_ by the mean of the two replicates. Next, the local noise estimate SD_diff_ (for each frame duration) was correlated with the tumor RF value bias, obtained by taking the difference between the reference scan and the noisy scan (RF_diff_ = RF_long_—RF_short_). Similarly, the local noise estimate COV_diff_ (for each frame duration) was correlated with the tumor RF value ratio, obtained by taking the ratio between the reference scan and the noisy scan (RF_ratio_ = RF_long_ / RF_short_) to take into account the percentage difference.

From each of the 10 replicates per scan duration, the median RF value was used and plotted as a function of image noise (either expressed as SD or COV as explained above). Next, a regression model was fitted using the variability of the feature values over the 10 replicates as a weighting factor. The following regressions were tested for each of the correctable features:

1. Linear, only slope (intercept = 0)
2. Linear, only intercept (fixed slope = 1)
3. Linear, with slope and intercept
4. Exponential
  - Model 1: $y=ae^{-bx}$
  - Model 2: $y=e^{a(x-b)}$
5. Sum of two exponentials: $y=ae^{-bx}+ce^{-dx}$
6. Reciprocal: $y=\frac{1}{a+bx}$
7. Double exponential
  - Model 1: $y=ae^{{-e}^{-b(x-c)}}$
  - Model 2: $y=e^{\left(\frac{a}{b}(1-e^{bx})\right)}$
8. Sigmoid/logistic
  - Model 1: $y=\frac{a}{1+be^{-cx}}$
  - Model 2: $y={a(1+(b-1)e^{-c(x-d)})}^{\frac{1}{1-b}}$
9. Power law
  - Model 1: $y=a(1-b^{x})$
  - Model 2: $y=ax^{b}$
10. Rational
  - Model 1: $\frac{ax}{b+x}$
  - Model 2: $\frac{a+bx}{1+cx}$

For linear regression models, the weighted least squares (WLS) method was used for estimating the parameters (slope with or without intercept) with the statsmodels (version 0.11) Python package [34], and for non-linear regression Levenberg–Marquardt algorithm with weighted input was used for curve-fitting with the *curve_fit* function from the scipy optimize (version 1.4.1) Python package [35]. The function *fsolve* from the same package was used to find the intercept for the linear model with a fixed slope of 1, since this functionality was not available in the statsmodels package.

The Akaike information criteria (AIC) was used to determine the best model (i.e., lowest AIC value). Then, the selected model was used to predict the ideal RF value, and the SDM was recalculated to check if the bias was compensated, i.e., whether the SDM increased and if this new SDM was reclassified as good or excellent.

*Feature correction for different reconstructions*. Modeling was done for EARL1 and EARL2 reconstructed scans and the corrected RF were calculated for each reconstruction. To explore if the correction parameters were in agreement between the different reconstruction protocols, the fit parameters from EARL1 were applied to the EARL2 data. Then, the resulting corrected RF were compared to those resulting from using EARL2 fit parameters and the percentage increase in SDM after feature correction was calculated.

## Results

### Analysis of precision

**Fig 3:**
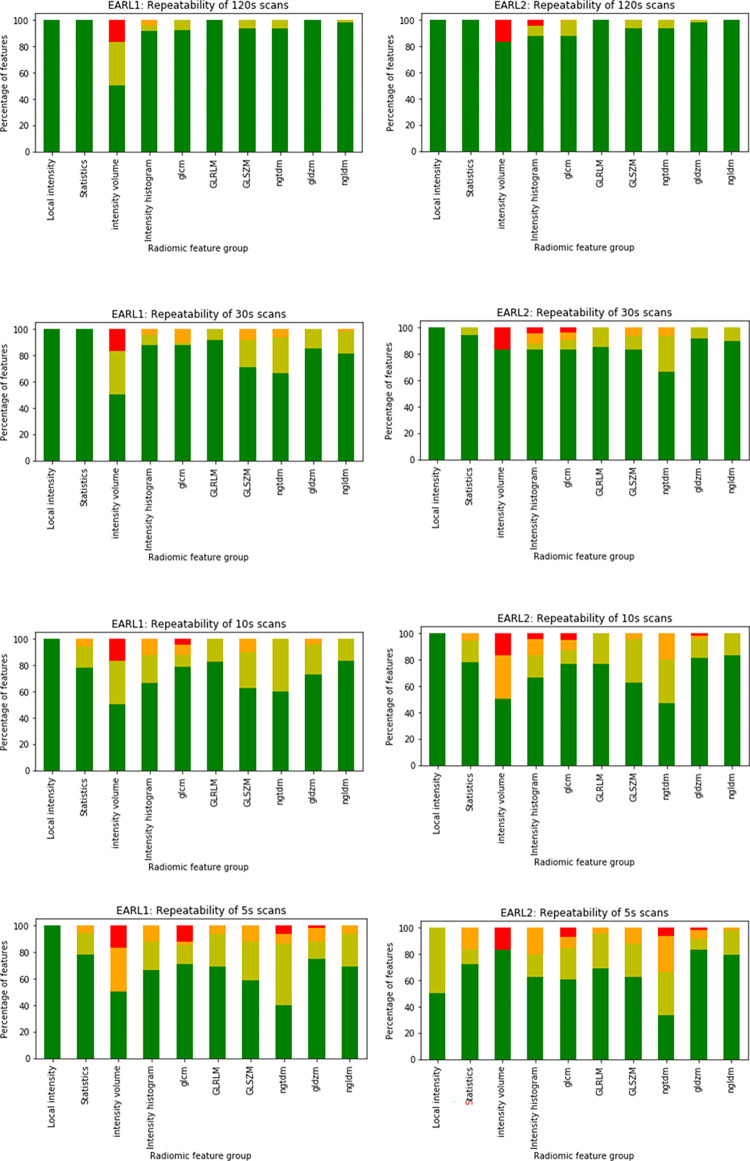
Percentage of features in each ICC category (dark green: Excellent ICC, light green: Good ICC, orange: Moderate ICC, red: Poor ICC) per feature group for different scan durations (from top to bottom: 120 s, 30 s, 10 s, 5 s) for EARL1 (left) and EARL2 (right) reconstructed images.

For EARL1 images, 95% of the features in the 120s scan duration had an excellent ICC, with the percentage decreasing at shorter durations: 85% for 30 s, 76% for 10 s, and 68% for 5 s. Similar results were obtained using the EARL2 reconstruction (S2 Table and Fig 3).

### Analysis of accuracy

**Fig 4:**
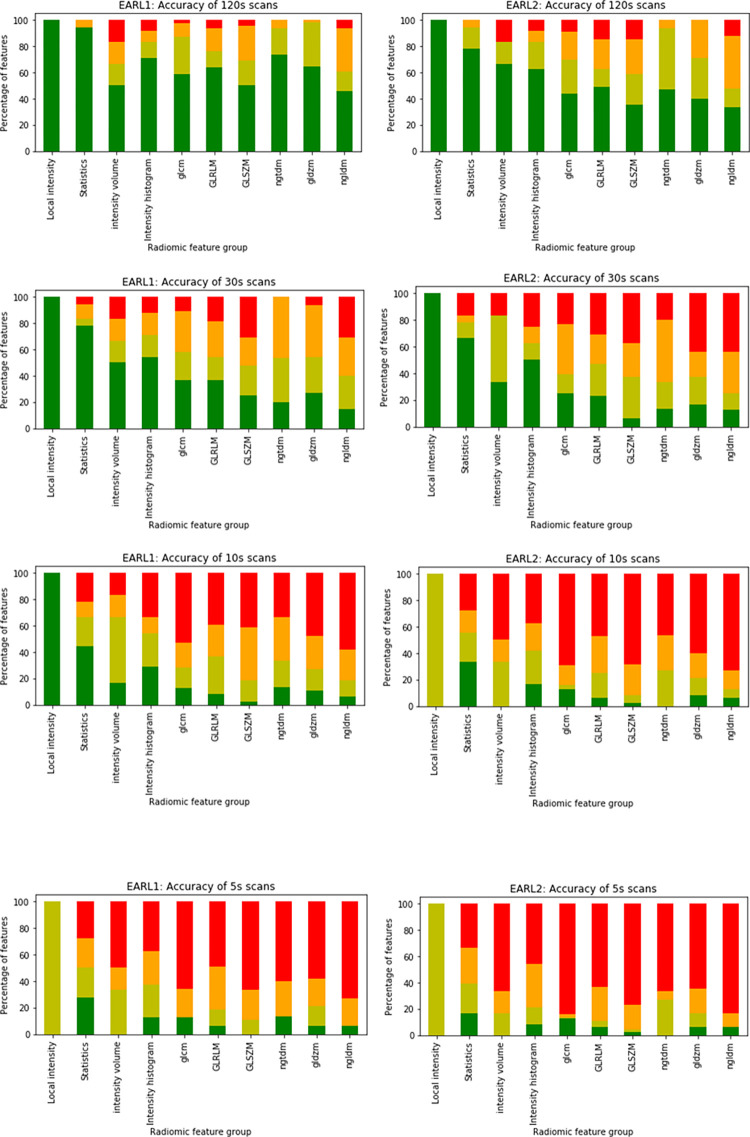
Percentage of features in each SDM category (dark green: Excellent SDM, light green: Good SDM, orange: Moderate SDM, red: Poor SDM) per feature group for different scan durations (from top to bottom: 120 s, 30 s, 10 s, 5 s) for EARL1 (left) and EARL2 (right) reconstructed images.

Shorter scan EARL1 reconstructed images showed significantly better accuracy when compared to EARL2 reconstructed images. The percentage of features with excellent SDM was the highest for the 120 s scans and decreased for the shorter scan duration images, with the lowest for the 5 s scans. The percentages of 120 s scan features yielding an excellent similarity distance from the 40-minute reference scan were 60.66%, and 45.49% for EARL1 and EARL2 reconstructions respectively, and they reduced to 9.01% and 8.13% correspondingly for 5 s scans. The number of features belonging to each SDM category is given in S3 Table. The percentage of features in each SDM category per feature group is shown in Fig 4.

### Radiomic feature correctability

**Fig 5:**
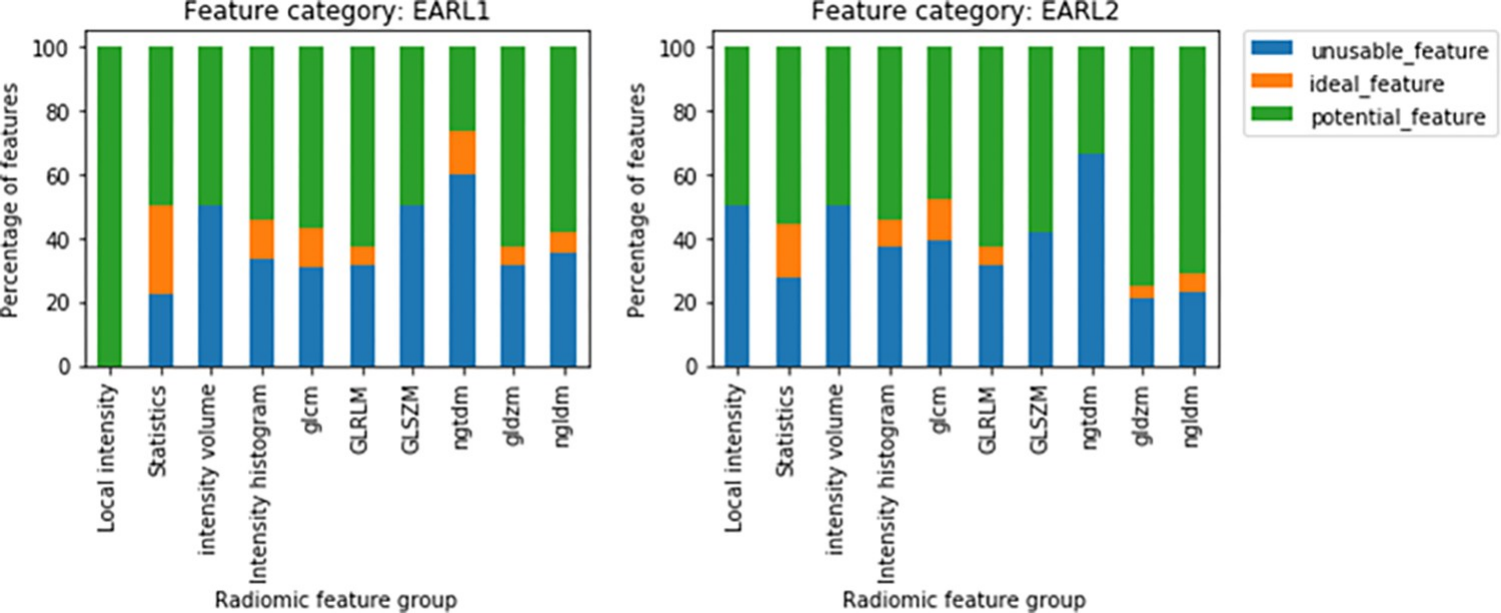
Percentage of features identified as unusable, ideal, and potential features per feature group for EARL1 (left) and EARL2 (right) reconstructed images.

Overall, 156 (34%) features in EARL1 and 158 features in EARL2 have been found to have low repeatability (ICC < 0.9), while only 41 (9%) of the features in EARL1 and 35 features in EARL2 showed an ideal performance with excellent ICC (ICC ≥ 0.9) and excellent SDM (SDM ≥ 0.9). The remaining 258 (57%) features in EARL1 and 262 features in EARL2 are found to have high repeatability (ICC ≥ 0.9) but poor accuracy (SDM < 0.9) in the presence of noise, and they were therefore checked for correctability. The percentage of features grouped into these three categories per feature group is shown in Fig 5. The percentage of features with excellent ICC and SDM per feature group are shown in S1 and S2 Figs. An example for a feature in each of these categories, showing the feature change with respect to the scan duration, is shown in S3 Fig.

The number of features passing each of the tests performed to check the feature correctability, per reconstruction protocol is shown in S4 Fig.

### Feature correction

After feature correction, the SDM improved for all the features when the most appropriate correction method was applied. The models reciprocal, double exponential (model 2), exponential (model 2), and power (model 1) worked best for estimating RF_ratio_ using COV_diff_ and the models linear (with slope and intercept), double exponential (model 1), power (model 1 and 2) logistic (model 1), rational (model 1) worked best for estimating RF_diff_ using SD_diff_. The best fits were chosen as per AIC and their frequency as the best choice for each of the variable pairs are listed in the S4 Table.

**Fig 6:**
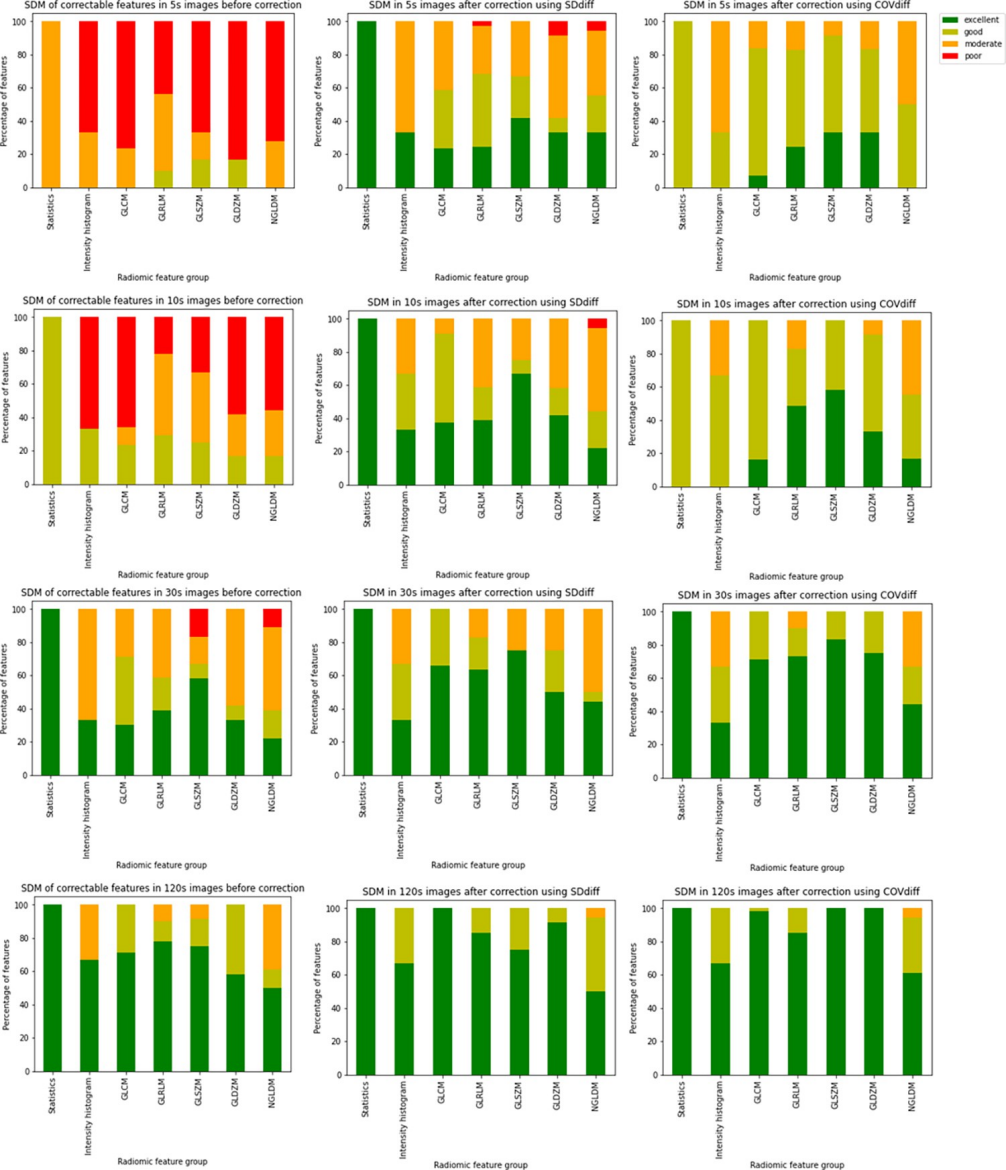
SDM of corrected features in EARL1 reconstructed images. From left to right: SDM before correction, SDM after correction using SD_diff_, and SDM after correction using COV_diff_. From top to bottom: SDM for different scan durations: 5 s, 10 s, 30 s, 120 s.

The results of SDM improvement due to noise-induced bias reduction have been restricted to the most noisy 5s scans. For the EARL1 reconstructed images, the SDM of 30 features improved to excellent and 7 features to good by correcting the RF_diff_ using SD_diff_ while that of 13 features improved to excellent and 29 to good by correcting the RF_ratio_ using COV_diff_. Fig 6 shows the SDM before and after correction using the two techniques for the different scan durations. For 10 features, both the correction techniques resulted in an excellent SDM, and either technique resulted in a good SDM for 31 features. In total, the SDM of 53 (i.e., 30+13+10) features improved to excellent and 67 (i.e., 7+29+31) improved to good, out of 143 correctable features. Overall, the mean percentage increase in SDM after feature correction using SD_diff_ was of 94.96. Using COV_diff_ on the other hand, resulted in a 111.51% increase in SDM on average for all the features.

For the EARL2 reconstruction, a comparison of the results obtained by using the same fit parameters in EARL1 reconstruction and by selecting the best fit for EARL2 reconstructed data for correcting 126 features using regression is shown in Table 2. In the first case, the percentage increase when using SD_diff_ and COV_diff_ were 131 and 222 respectively and in the latter case 196 and 237 correspondingly.

**Table 2:** Comparison of EARL2 results obtained by using the same fit parameters in EARL1 reconstruction with those by selecting the best fit for EARL2 reconstructed data using regression.

SDM improvement	Correction technique	Number of features showing improvement	
		Using fit parameters from EARL1	EARL2 model
Excellent	SD_diff_ only	19	14
	COV_diff_ only	8	6
	both SD_diff_ and COV_diff_	4	13
Good	SD_diff_ only	1	8
	COV_diff_ only	42	31
	both SD_diff_ and COV_diff_	18	36

## Discussion

Simulation of different noise levels, e.g., those seen in low photon count tracers, by varying the scan duration has shown that even though the noise-induced variability between the replicates remained low for several features, there is a systematic bias in the radiomic feature values of images with low SNR. For a feature to have a predictive value and to be useful in the oncological clinical studies, it is desired that the feature value depends on the tumor characteristics only and not on noise. This implies that radiomics analysis can only be used in high count studies where the image quality is high or after features are corrected for noise-induced variability and/or biases. In order to correct for noise-induced bias, we have evaluated the feasibility of using several regression models describing the relationship between the feature value and the local noise estimate.

In this study, a large number of features have shown reasonably high repeatability in images with poor SNR for both EARL1 and EARL2 reconstructions. Features from EARL1 reconstructed images have comparatively shown better repeatability most probably because of increased smoothing with Gaussian filter, as Gaussian smoothing is known to reduce the image intensity variations between voxels, making the image more homogenous. While this is useful for image noise reduction, it is expected to also remove tumor heterogeneity details and, consequently, some PET textural features may no longer have a meaningful predictive value. Hence, there is always a trade-off between noise and image resolution. However, Pfaehler et. al has shown that an increase in the Gaussian smoothing do not necessarily eliminate all important heterogeneity information [19]. Also, several studies have come up with different noise reduction techniques while still preserving spatial resolution [36, 37]. Therefore, image noise reduction using different filtering techniques should be further explored to reduce the noise-induced variability in the radiomic features derived from poor SNR images.

Even though several features were stable with high repeatability, they showed a systematic noise dependent bias. Shorter EARL1 reconstructed images showed better accuracy when compared to EARL2 reconstructed images. We have shown that it is possible to characterize the trend of the feature bias with local image noise. Using both SD_diff_ and COV_diff_ from the difference image resulted in bias reduction, with COV_diff_ providing overall better results than SD_diff_, although the selection of either SD_diff_ or COV_diff_ is feature dependent in some cases. In addition, we see that the feature bias trend with local image noise was similar between the two EARL compliant protocols pointing to possible compatibility between the different versions of the recommended reconstruction settings for harmonization. Moreover, in addition to the use of the local noise information, we also evaluated the possibility of using the background noise for feature bias trend characterization. However, the outcome of this approach is not reported here, as it yielded poor results, probably because the PET textural features are highly sensitive to intensity variation between neighboring voxels and background noise estimates may not completely capture local intensity variations [38]. Our assumption is that a single combination of two frames would provide a sufficient estimate of the local noise to correct for noise-induced bias in features with high precision (high ICC and low COV) and low accuracy. While it is possible to average over multiple combinations of two frames with the SD describing the variability, in reality, it is practical to perform only two split reconstructions due to time limitation and computational complexity. Furthermore, performing multiple splits on low count PET studies, such as ^89^Zr-Immuno-PET, might result in high noise-induced variability between the different splits making it unsuitable for noise-induced bias mitigation.

The possibility to characterize the trend of the feature bias with local image noise opens the door to a wide range of situations where noise is a dominant factor. While this study was done based on the noise conditions seen in ^89^Zr-Immuno-PET, the concept can be easily expanded to other tracers with poor count statistics and even to high count tracers, such as ^18^F-FDG, with the purpose to reduce either scan duration or the injected activity. Another possible application could be to reduce the impact of feature bias due to noise based on the sensitivity of PET detectors, thus eliminating PET/CT system bias which can be especially useful in multicenter studies. Moreover, we have explored two different recommended reconstruction protocols (EARL standards 1 and 2) yielding similar contrast recoveries for harmonization in multicenter studies to check if our methodology will be applicable for different reconstruction settings [39].

One possible limitation of this study was that we used only nine tumor inserts, all of them with fairly high uptake (the lowest TBR was 2.5) and relatively big size. Yet, smaller lesions may occur in clinical data. However, Hatt et. al has recommended to apply radiomics analyses to lesions larger than 10 cm^3^ [40]. Moreover, Pfaehler et. al has shown that bigger spheres and higher activity uptakes yielded better repeatable features than smaller spheres and those with a lower activity uptake [19]. In clinical settings, there could be tumors with a different uptake pattern that was not represented in our study, smaller tumors, and tumors with very low uptake (even lower than the surrounding tissues), all this affecting the repeatability of RF. However, the exclusion of these confounding factors and availability of ground truth values is exactly the advantage of a phantom study and we have shown the feasibility of identifying robust and correctable features, in addition to noise-induced bias mitigating strategies. An approximation of the ideal image was obtained by performing a long, almost noise-free acquisition of 40 minutes which is 20 times longer than performed in clinic (2 min per bed position). While this reference image is an approximated ground truth which may suffer from very minimal noise, the noise level is low enough that it can be considered as ground truth. This was verified by the very minimal COV in the background, which for example, ranged between 3.33% and 6.47% for the three different TBR in the EARL1 reconstructed images. Although this is strictly not noise free, we do assume that the noise level is sufficiently low to approximate a noise-free scan. Moreover, the 3D printed tumor inserts helped in simulating realistic conditions as opposed to spherical phantom inserts, and Pfaehler et al. have validated previously that radiomic features obtained with the 3D inserts are representative of true NSCLC tumour lesions showing an excellent correspondence between phantom and clinical data feature values [19].

Another limitation is that we only used limited regression techniques for our hypothesis to describe the noise-induced bias. More complex models might give better results, although with a higher risk to overfit the data. Since substantial bias reduction for the correctable features was achieved with simple methods, and this was not the main aim of the study, it was decided to not include more complex approaches. Yet, it will be easy to extend the approach to include them in future studies. For now, we were able to show that models using a noise-estimate as input can be used to reduce the noise-induced bias under very poor signal conditions.

While in our study we only analyzed the impact of the noise on the repeatability and accuracy of the radiomic features, the noise present in the images also makes segmentation a difficult task, leading to wide inter-observer variability. These discrepancies in tumor segmentation can also affect the repeatability and accuracy of the radiomic features derived from low SNR images.

## Conclusion

Several radiomic features derived from low SNR images have high repeatability, but many of them show a substantial noise-induced bias. For a feature to have a predictive value, we want the feature value to depend on the tumor characteristics only and not on noise. We have shown that it is possible to correct the radiomic feature noise-induced bias with simple models using the local image noise estimate as input. These models may allow the use of radiomics analysis for low count studies, such as with ^89^Zr-labelled mAbs, or to develop a clinical radiomics model in patient studies obtained on different PET systems with substantial differences in image quality e.g., because of differences in sensitivity.

## Supporting information

S1 Fig Percentage of features with excellent ICC per feature group in EARL1 and EARL2 reconstructed images for scan durations of 120s and 5s. (TIF)

S2 Fig Percentage of features with excellent SDM per feature group in EARL1 and EARL2 reconstructed images for scan durations of 120s and 5s. (TIF)

S3 Fig Example for ideal feature, potential feature, and unusable feature (left to right) showing the feature behaviour change with scan duration. Note how the ideal feature and potential feature have smaller error bars unlike the unusable feature. Their feature values are also consistent for all scan durations, or in other words the lines do not cross each other and stay parallel to each other, while the opposite is true for the unusable feature. Also note how the ideal feature has the same value for all scan durations, or in other words the lines are straight. On the other hand, there is systematic bias in the potential feature with scan duration, or in other words there is a (linear/non-linear) relationship between the feature value and the scan duration.
(TIF)

S4 Fig Number of features passing each of the tests performed to check the radiomic feature correctability, per reconstruction protocol. (TIF)

S1 Table List of radiomic features calculated as per IBSI guidelines. Note: The feature Dependence count percentage belonging to the family NGLDM (2Davg, 2Dmrg, 3Dmrg) was always 1 irrespective of the tumor. So, it is excluded in the analysis, which leaves 455 features.
(DOCX)

S2 Table Number of features belonging to each ICC category. (DOCX)

S3 Table Number of features belonging to each SDM category. (DOCX)

S4 Table Best fits chosen as per AIC and their frequency for the dependent-independent variable pairs: RF_ratio_ vs COV_diff_ and RF_diff_ vs SD_diff_, in EARL1 and EARL2 reconstructed data. (DOCX)
