# Supplementary figures and images for: Mitigation of noise-induced bias of PET radiomic features
Source: PLoS One. 2022 Aug 25;17(8):e0272643. doi: 10.1371/journal.pone.0272643 (PMC9409510; doi:10.1371/journal.pone.0272643)

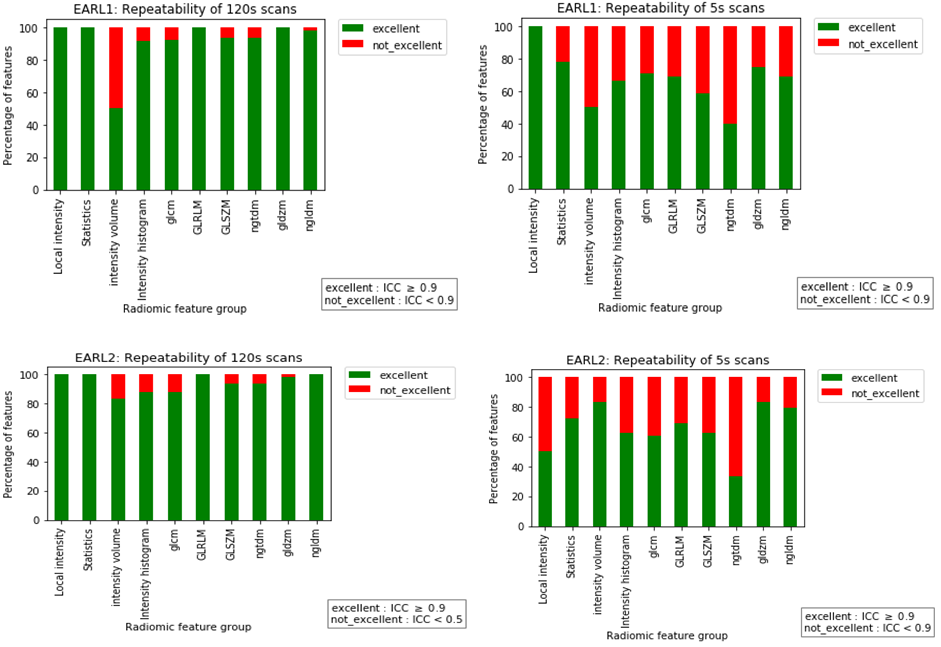

Supplement: S1 Fig — (TIF) [file pone.0272643.s001.tif]

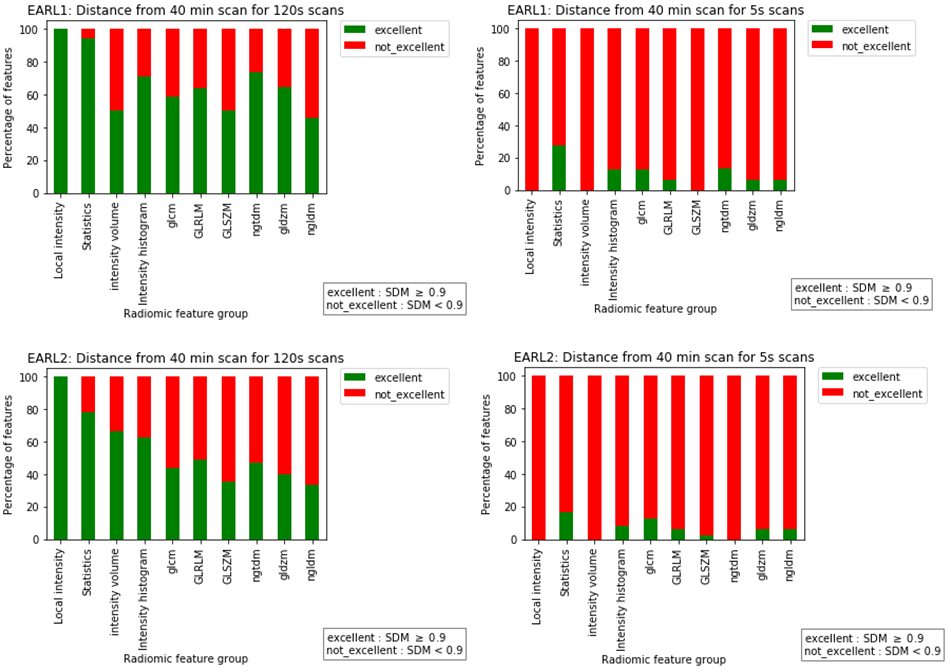

Supplement: S2 Fig — (TIF) [file pone.0272643.s002.tif]

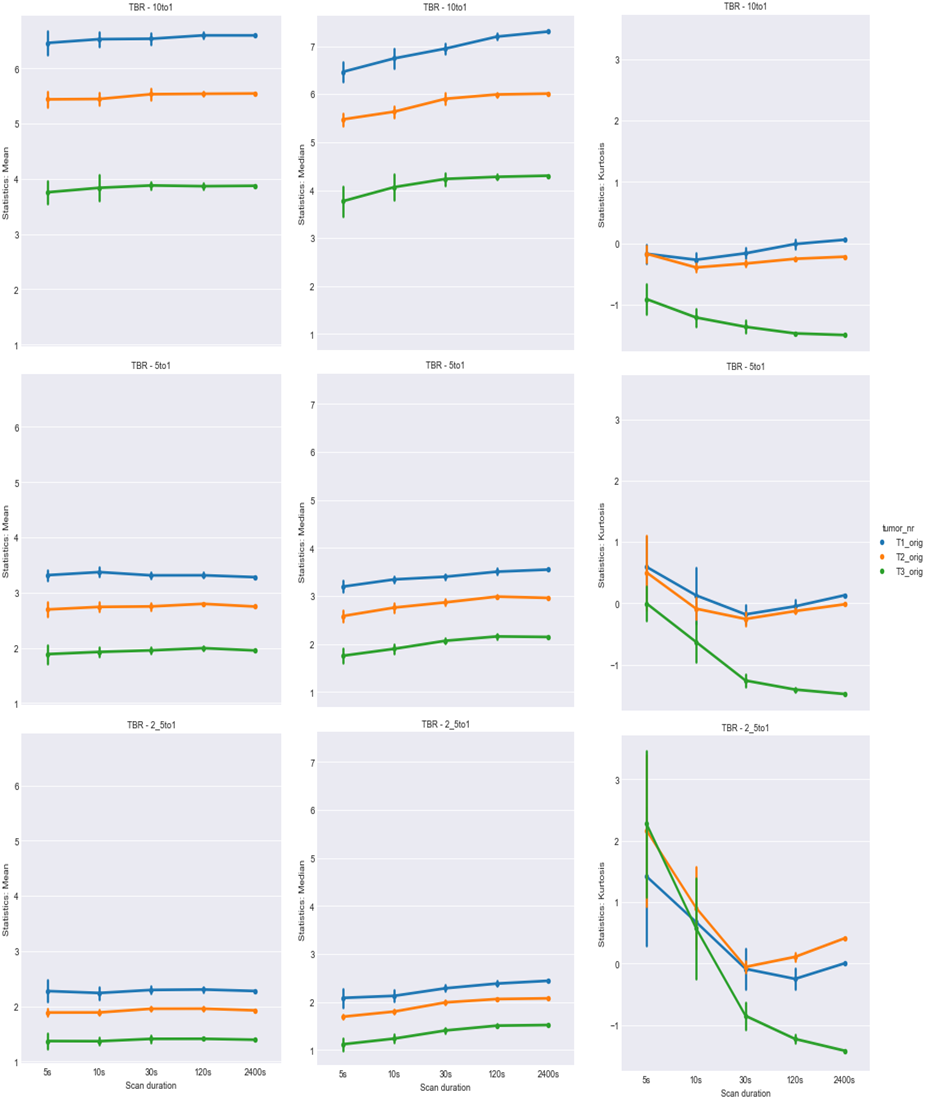

Supplement: S3 Fig — Example for ideal feature, potential feature, and unusable feature (left to right) showing the feature behaviour change with scan duration. Note how the ideal feature and potential feature have smaller error bars unlike the unusable feature. Their feature values are also consistent for all scan durations, or in other words the lines do not cross each other and stay parallel to each other, while the opposite is true for the unusable feature. Also note how the ideal feature has the same value for all scan durations, or in other words the lines are straight. On the other hand, there is systematic bias in the potential feature with scan duration, or in other words there is a (linear/non-linear) relationship between the feature value and the scan duration. (TIF) [file pone.0272643.s003.tif]

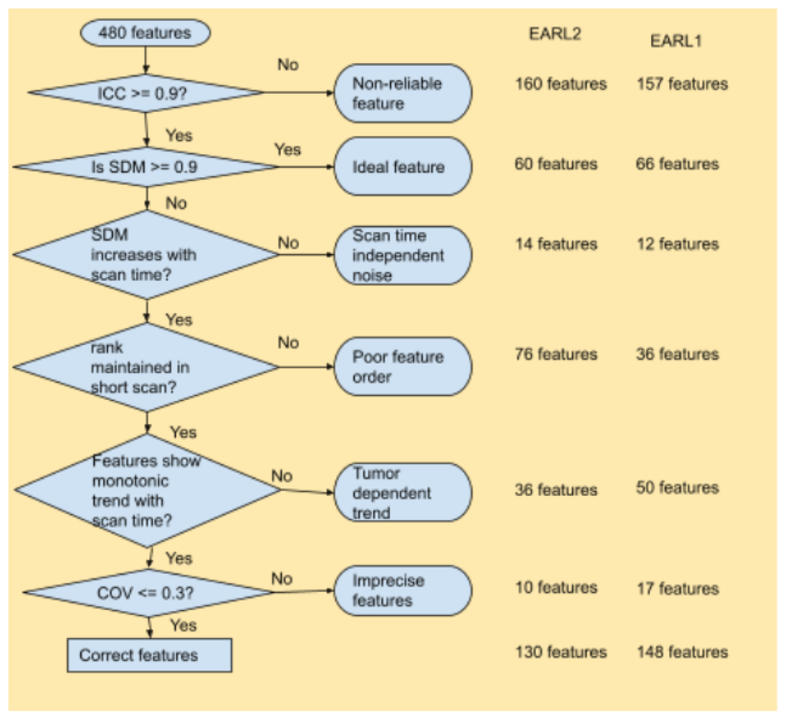

Supplement: S4 Fig — (TIF) [file pone.0272643.s004.tif]
