# Supplementary material for: Mitigation of noise-induced bias of PET radiomic features
Source: PLoS One. 2022 Aug 25;17(8):e0272643. doi: 10.1371/journal.pone.0272643 (PMC9409510; doi:10.1371/journal.pone.0272643)
Supplement: S1 Table — Note: The feature Dependence count percentage belonging to the family NGLDM (2Davg, 2Dmrg, 3Dmrg) was always 1 irrespective of the tumor. So, it is excluded in the analysis, which leaves 455 features. (DOCX) [file pone.0272643.s005.docx]

| **feature_group** | **feature_name** |
| --- | --- |
| Local intensity | local intensity peak |
| Local intensity | global intensity peak |
| Statistics | mean |
| Statistics | median |
| Statistics | 10th percentile |
| Statistics | 90th percentile |
| Statistics | Root mean |
| intensity volume | int at vol fraction 10 |
| Intensity histogram | mean |
| Intensity histogram | median |
| Intensity histogram | 10th percentile |
| Intensity histogram | 90th percentile |
| Intensity histogram | mode |
| glcmFeatures2Davg | joint maximum |
| glcmFeatures2Davg | joint average |
| glcmFeatures2Davg | joint entropy |
| glcmFeatures2Davg | difference average |
| glcmFeatures2Davg | sum average |
| glcmFeatures2Davg | dissimilarity |
| glcmFeatures2Davg | inverse difference |
| glcmFeatures2Davg | inverse difference moment |
| glcmFeatures2Davg | autocorrelation |
| glcmFeatures2DDmrg | joint maximum |
| glcmFeatures2DDmrg | joint average |
| glcmFeatures2DDmrg | difference average |
| glcmFeatures2DDmrg | difference entropy |
| glcmFeatures2DDmrg | sum average |
| glcmFeatures2DDmrg | dissimilarity |
| glcmFeatures2DDmrg | inverse difference |
| glcmFeatures2DDmrg | inverse difference moment |
| glcmFeatures2DDmrg | autocorrelation |
| glcmFeatures2Dmrg | joint average |
| glcmFeatures2Dmrg | difference average |
| glcmFeatures2Dmrg | sum average |
| glcmFeatures2Dmrg | dissimilarity |
| glcmFeatures2Dmrg | inverse difference |
| glcmFeatures2Dmrg | inverse difference moment |
| glcmFeatures2Dmrg | autocorrelation |
| glcmFeatures2Dvmrg | joint average |
| glcmFeatures2Dvmrg | difference average |
| glcmFeatures2Dvmrg | difference entropy |
| glcmFeatures2Dvmrg | sum average |
| glcmFeatures2Dvmrg | dissimilarity |
| glcmFeatures2Dvmrg | inverse difference |
| glcmFeatures2Dvmrg | inverse difference moment |
| glcmFeatures2Dvmrg | autocorrelation |
| glcmFeatures3Davg | joint average |
| glcmFeatures3Davg | difference average |
| glcmFeatures3Davg | difference entropy |
| glcmFeatures3Davg | sum average |
| glcmFeatures3Davg | dissimilarity |
| glcmFeatures3Davg | inverse difference |
| glcmFeatures3Davg | inverse difference normalised |
| glcmFeatures3Davg | inverse difference moment |
| glcmFeatures3Davg | inverse variance |
| glcmFeatures3Davg | autocorrelation |
| glcmFeatures3DWmrg | joint average |
| glcmFeatures3DWmrg | difference average |
| glcmFeatures3DWmrg | difference entropy |
| glcmFeatures3DWmrg | sum average |
| glcmFeatures3DWmrg | dissimilarity |
| glcmFeatures3DWmrg | inverse difference |
| glcmFeatures3DWmrg | inverse difference moment |
| glcmFeatures3DWmrg | inverse variance |
| glcmFeatures3DWmrg | autocorrelation |
| GLRLMFeatures2Davg | Low grey level run emphasis |
| GLRLMFeatures2Davg | High grey level run emphasis |
| GLRLMFeatures2Davg | Short-run low grey level emphasis |
| GLRLMFeatures2Davg | Short-run high grey level emphasis |
| GLRLMFeatures2Davg | Long run high grey level emphasis |
| GLRLMFeatures2Davg | Grey level non-uniformity |
| GLRLMFeatures2Davg | Run-length non-uniformity |
| GLRLMFeatures2Davg | Run-length non-uniformity normalized |
| GLRLMFeatures2Davg | Run percentage |
| GLRLMFeatures2Davg | Run-length variance |
| GLRLMFeatures2DDmrg | Short-run emphasis |
| GLRLMFeatures2DDmrg | Low grey level run emphasis |
| GLRLMFeatures2DDmrg | High grey level run emphasis |
| GLRLMFeatures2DDmrg | Short-run low grey level emphasis |
| GLRLMFeatures2DDmrg | Short-run high grey level emphasis |
| GLRLMFeatures2DDmrg | Long run high grey level emphasis |
| GLRLMFeatures2DDmrg | Grey level non-uniformity |
| GLRLMFeatures2DDmrg | Run-length non-uniformity |
| GLRLMFeatures2DDmrg | Run-length non-uniformity normalized |
| GLRLMFeatures2DDmrg | Run percentage |
| GLRLMFeatures2DWmrg | Low grey level run emphasis |
| GLRLMFeatures2DWmrg | High grey level run emphasis |
| GLRLMFeatures2DWmrg | Short-run low grey level emphasis |
| GLRLMFeatures2DWmrg | Short-run high grey level emphasis |
| GLRLMFeatures2DWmrg | Long run high grey level emphasis |
| GLRLMFeatures2DWmrg | Grey level non-uniformity |
| GLRLMFeatures2DWmrg | Run-length non-uniformity |
| GLRLMFeatures2DWmrg | Run-length non-uniformity normalized |
| GLRLMFeatures2DWmrg | Run percentage |
| GLRLMFeatures2Dvmrg | Short-run emphasis |
| GLRLMFeatures2Dvmrg | Low grey level run emphasis |
| GLRLMFeatures2Dvmrg | High grey level run emphasis |
| GLRLMFeatures2Dvmrg | Short-run low grey level emphasis |
| GLRLMFeatures2Dvmrg | Short-run high grey level emphasis |
| GLRLMFeatures2Dvmrg | Long run high grey level emphasis |
| GLRLMFeatures2Dvmrg | Grey level non-uniformity |
| GLRLMFeatures2Dvmrg | Run-length -non-uniformity |
| GLRLMFeatures2Dvmrg | Run-length non-uniformity normalized |
| GLRLMFeatures2Dvmrg | Run percentage |
| GLRLMFeatures3Davg | Short-run emphasis |
| GLRLMFeatures3Davg | Low grey level run emphasis |
| GLRLMFeatures3Davg | High grey level run emphasis |
| GLRLMFeatures3Davg | Short-run low grey level emphasis |
| GLRLMFeatures3Davg | Short-run high grey level emphasis |
| GLRLMFeatures3Davg | Long run low grey level emphasis |
| GLRLMFeatures3Davg | Long run high grey level emphasis |
| GLRLMFeatures3Davg | Grey level non-uniformity |
| GLRLMFeatures3Davg | Run-length non-uniformity |
| GLRLMFeatures3Davg | Run-length non-uniformity normalized |
| GLRLMFeatures3Davg | Run percentage |
| GLRLMFeatures3Dmrg | Low grey level run emphasis |
| GLRLMFeatures3Dmrg | High grey level run emphasis |
| GLRLMFeatures3Dmrg | Short-run low grey level emphasis |
| GLRLMFeatures3Dmrg | Short-run high grey level emphasis |
| GLRLMFeatures3Dmrg | Long run low grey level emphasis |
| GLRLMFeatures3Dmrg | Long run high grey level emphasis |
| GLRLMFeatures3Dmrg | Grey level non-uniformity |
| GLRLMFeatures3Dmrg | Run-length non-uniformity |
| GLRLMFeatures3Dmrg | Run-length non-uniformity normalized |
| GLRLMFeatures3Dmrg | Run percentage |
| GLSZMFeatures2Davg | Low grey-level zone emphasis |
| GLSZMFeatures2Davg | High grey-level zone emphasis |
| GLSZMFeatures2Davg | Small zone high grey level emphasis |
| GLSZMFeatures2Davg | Grey level non-uniformity GLSZM |
| GLSZMFeatures2Davg | Zone size non-uniformity normalized |
| GLSZMFeatures2Davg | Zone percentage GLSZM |
| GLSZMFeatures2Davg | Zone size entropy |
| GLSZMFeatures2Dvmrg | Low grey-level zone emphasis |
| GLSZMFeatures2Dvmrg | High grey-level zone emphasis |
| GLSZMFeatures2Dvmrg | Small zone high grey level emphasis |
| GLSZMFeatures2Dvmrg | Grey level non-uniformity GLSZM |
| GLSZMFeatures2Dvmrg | Zone size non-uniformity normalized |
| GLSZMFeatures2Dvmrg | Zone percentage GLSZM |
| GLSZMFeatures3D | High grey level zone emphasis |
| GLSZMFeatures3D | Small zone high grey level emphasis |
| GLSZMFeatures3D | Zone percentage GLSZM |
| GLSZMFeatures3D | Zone size entropy |
| ngtdmFeatures2Dmrg | coarseness |
| ngtdmFeatures2Dmrg | contrast |
| ngtdmFeatures3D | coarseness |
| ngtdmFeatures3D | contrast |
| gldzmFeatures2Davg | small distance emphasis GLDZM |
| gldzmFeatures2Davg | Large distance emphasis GLDZM |
| gldzmFeatures2Davg | Low grey level zone emphasis GLDZM |
| gldzmFeatures2Davg | High grey level zone emphasis GLDZM |
| gldzmFeatures2Davg | Small distance high grey level emphasis GLDZM |
| gldzmFeatures2Davg | Large distance low grey level emphasis GLDZM |
| gldzmFeatures2Davg | Grey level non uniformity GLDZM |
| gldzmFeatures2Davg | Zone distance non uniformity GLDZM |
| gldzmFeatures2Davg | Zone percentage GLDZM |
| gldzmFeatures2Davg | Zone distance variance GLDZM |
| gldzmFeatures2Davg | Zone distance entropy GLDZM |
| gldzmFeatures2Dmrg | small distance emphasis GLDZM |
| gldzmFeatures2Dmrg | Large distance emphasis GLDZM |
| gldzmFeatures2Dmrg | Low grey level zone emphasis GLDZM |
| gldzmFeatures2Dmrg | High grey level zone emphasis GLDZM |
| gldzmFeatures2Dmrg | Small distance low grey level emphasis GLDZM |
| gldzmFeatures2Dmrg | Small distance high grey level emphasis GLDZM |
| gldzmFeatures2Dmrg | Large distance low grey level emphasis GLDZM |
| gldzmFeatures2Dmrg | Grey level non-uniformity GLDZM |
| gldzmFeatures2Dmrg | Zone distance non-uniformity GLDZM |
| gldzmFeatures2Dmrg | Zone percentage GLDZM |
| gldzmFeatures2Dmrg | Zone distance variance GLDZM |
| gldzmFeatures3D | Large distance emphasis GLDZM |
| gldzmFeatures3D | High grey-level zone emphasis GLDZM |
| gldzmFeatures3D | Small distance high grey level emphasis GLDZM |
| gldzmFeatures3D | Zone percentage GLDZM |
| gldzmFeatures3D | Zone distance variance GLDZM |
| ngldmFeatures2Davg | Low dependence emphasis |
| ngldmFeatures2Davg | Low grey level count emphasis |
| ngldmFeatures2Davg | High grey level count emphasis |
| ngldmFeatures2Davg | Low dependence high grey level emphasis |
| ngldmFeatures2Davg | Grey level non-uniformity |
| ngldmFeatures2Davg | Dependence count non-uniformity |
| ngldmFeatures2Davg | Dependence count non-uniformity normalized |
| ngldmFeatures2Davg | Dependence count entropy |
| ngldmFeatures2Dmrg | Low dependence emphasis |
| ngldmFeatures2Dmrg | Low grey level count emphasis |
| ngldmFeatures2Dmrg | High grey level count emphasis |
| ngldmFeatures2Dmrg | Low dependence high grey level emphasis |
| ngldmFeatures2Dmrg | Grey level non-uniformity |
| ngldmFeatures2Dmrg | Dependence count non-uniformity |
| ngldmFeatures2Dmrg | Dependence count non-uniformity normalized |
| ngldmFeatures3Dmrg | Low dependence emphasis |
| ngldmFeatures3Dmrg | Low grey level count emphasis |
| ngldmFeatures3Dmrg | High grey level count emphasis |
| ngldmFeatures3Dmrg | Low dependence high grey level emphasis |
| ngldmFeatures3Dmrg | Grey level non-uniformity |
| ngldmFeatures3Dmrg | Dependence count non-uniformity |
| ngldmFeatures3Dmrg | Dependence count non-uniformity normalized |
| ngldmFeatures3Dmrg | Dependence count entropy |
