# Supplementary material for: Mitigation of noise-induced bias of PET radiomic features
Source: PLoS One. 2022 Aug 25;17(8):e0272643. doi: 10.1371/journal.pone.0272643 (PMC9409510; doi:10.1371/journal.pone.0272643)
Supplement: S2 Table — (DOCX) [file pone.0272643.s006.docx]

| ICC Category | 120 s | | 30 s | | 10 s | | 5 s | |
| --- | --- | --- | --- | --- | --- | --- | --- | --- |
|  | EARL1 | EARL2 | EARL1 | EARL2 | EARL1 | EARL2 | EARL1 | EARL2 |
| Excellent | 433 | 428 | 388 | 388 | 346 | 340 | 310 | 304 |
| Good | 20 | 24 | 44 | 45 | 79 | 81 | 93 | 99 |
| Moderate | 1 | 1 | 22 | 14 | 22 | 23 | 31 | 38 |
| Poor | 1 | 2 | 1 | 8 | 8 | 11 | 21 | 14 |
