# Supplementary material for: Mitigation of noise-induced bias of PET radiomic features
Source: PLoS One. 2022 Aug 25;17(8):e0272643. doi: 10.1371/journal.pone.0272643 (PMC9409510; doi:10.1371/journal.pone.0272643)
Supplement: S3 Table — (DOCX) [file pone.0272643.s007.docx]

| SDM Category | 120 s | | 30 s | | 10 s | | 5 s | |
| --- | --- | --- | --- | --- | --- | --- | --- | --- |
|  | EARL1 | EARL2 | EARL1 | EARL2 | EARL1 | EARL2 | EARL1 | EARL2 |
| Excellent | 276 | 207 | 157 | 106 | 56 | 43 | 41 | 37 |
| Good | 94 | 100 | 96 | 87 | 88 | 53 | 38 | 25 |
| Moderate | 67 | 105 | 130 | 124 | 104 | 89 | 109 | 67 |
| Poor | 18 | 43 | 72 | 138 | 207 | 270 | 267 | 326 |
