# Supplementary material for: Mitigation of noise-induced bias of PET radiomic features
Source: PLoS One. 2022 Aug 25;17(8):e0272643. doi: 10.1371/journal.pone.0272643 (PMC9409510; doi:10.1371/journal.pone.0272643)
Supplement: S4 Table — (DOCX) [file pone.0272643.s008.docx]

| **Best fits chosen as per AIC** | **EARL1** | | **EARL2 (same features corrected as EARL1)** | | **EARL2 (separate features categorization)** | |
| --- | --- | --- | --- | --- | --- | --- |
|  | COV_diff_ | SD_diff_ | COV_diff_ | SD_diff_ | COV_diff_ | SD_diff_ |
| linear_with_intercept | 6 | 14 | 7 | 14 | 7 | 24 |
| linear_without_intercept | 1 | 0 | 1 | 1 | 1 | 1 |
| linear_with_fixed_slope_of_1 | 0 | 0 | 0 | 1 | 2 | 1 |
| reciprocal | 49 | 3 | 47 | 1 | 32 | 3 |
| double_exponential_1 | 0 | 17 | 0 | 22 | 0 | 12 |
| double_exponential_2 | 33 | 3 | 28 | 3 | 27 | 3 |
| exponential_1 | 1 | 4 | 0 | 3 | 0 | 3 |
| exponential_2 | 27 | 2 | 25 | 4 | 30 | 3 |
| sum_of_two_exponentials | 2 | 0 | 2 | 0 | 3 | 0 |
| power_func_1 | 20 | 27 | 20 | 28 | 17 | 17 |
| power_func_2 | 0 | 14 | 2 | 13 | 2 | 10 |
| logistic_1 | 4 | 28 | 6 | 16 | 6 | 19 |
| logistic_2 | 0 | 1 | 0 | 1 | 0 | 1 |
| Polyratio_1 | 1 | 26 | 7 | 19 | 5 | 19 |
| Polyratio_2 | 0 | 4 | 0 | 11 | 0 | 14 |
